# Health system efficiency in Germany: results of a pilot study to assess health system performance

**DOI:** 10.1093/eurpub/ckac131.024

**Published:** 2022-10-25

**Authors:** P Hengel, K Achstetter, M Blümel, V Schwarzbach, R Busse

**Affiliations:** Department of Health Care Management, Technische Universität Berlin, Berlin, Germany

## Abstract

**Background:**

Improved efficiency is one overall goal in WHO’s Health Systems Framework. Efficiency is an important dimension of health system performance assessment (HSPA). HSPA is used as a tool to monitor and evaluate the performance of health systems and to support evidence-based policymaking. In the pilot study for a first German HSPA, efficiency was assessed as one dimension.

**Methods:**

Indicators were selected based on a systematic search of established instruments in national and international HSPA initiatives. Criteria for the inclusion of indicators were data availability and international comparability. Where possible, indicators were evaluated in terms of their development over time (2000-2020), in comparison to eight European countries (e.g., Austria, Denmark, France), and regarding equity aspects (e.g., age, gender, region).

**Results:**

Eight indicators to assess the efficiency of the German health system were identified and analysed accordingly. They cover the pharmaceutical sector, outpatient and inpatient care, and system-wide efficiency. Trend analyses were possible for all indicators, and most were also suitable for international comparisons. Overall, results of the chosen indicators indicate a moderate health system efficiency. The volume of generics as share of all pharmaceuticals, e.g., was 83% in Germany in 2019 (country average: 54%) and has been steadily increasing since 2000. In contrast, expenses for pharmaceuticals overall rose from 1.4% of GDP in 2004 to 1.7% in 2019, whereas it declined from 1.3% to 1.1% on average in the other countries.

**Conclusions:**

Within this first pilot study, a systematic and comparative German HSPA measuring the efficiency of the German health system using eight predefined indicators was proven to be feasible. The results give insights into efficiency measurements across different sectors, e.g., pharmaceuticals, identify developments of efficiency over time, and can support evidence-based policymaking.

**Key messages:**

• In the pilot study for a first German HSPA, efficiency was evaluated using eight indicators covering pharmaceuticals, outpatient and inpatient care, and system-wide efficiency.

• Based on the available data, which allowed trend analyses for all indicators and comparisons to eight European countries for most indicators, Germany’s health system efficiency can still be improved.

